# Contrasting responses of water use efficiency to drought across global terrestrial ecosystems

**DOI:** 10.1038/srep23284

**Published:** 2016-03-17

**Authors:** Yuting Yang, Huade Guan, Okke Batelaan, Tim R. McVicar, Di Long, Shilong Piao, Wei Liang, Bing Liu, Zhao Jin, Craig T. Simmons

**Affiliations:** 1 School of the Environment, Flinders University, Adelaide, South Australia 5042, Australia; 2 CSIRO Land and Water, Canberra, ACT 2601, Australia; 3 National Centre for Groundwater Research and Training, Adelaide, South Australia 5042, Australia; 4 Australian Research Council Centre of Excellence for Climate System Science, Sydney, Australia; 5 State Key Laboratory of Hydroscience and Engineering, Department of Hydraulic Engineering, Tsinghua University, Beijing 100084, China; 6 Sino-French Institute for Earth System Science, College of Urban and Environmental Sciences, Peking University, Beijing 100871, China; 7 College of Tourism and Environmental Sciences, Shaanxi Normal University, Xi’an 710062, China; 8 Linze Inland River Basin Research Station, Laboratory of Heihe River Eco-Hydrology and Basin Science, Cold and Arid Regions Environmental and Engineering Research Institute, Chinese Academy of Sciences, Lanzhou 730000, China

## Abstract

Drought is an intermittent disturbance of the water cycle that profoundly affects the terrestrial carbon cycle. However, the response of the coupled water and carbon cycles to drought and the underlying mechanisms remain unclear. Here we provide the first global synthesis of the drought effect on ecosystem water use efficiency (WUE = gross primary production (GPP)/evapotranspiration (ET)). Using two observational WUE datasets (i.e., eddy-covariance measurements at 95 sites (526 site-years) and global gridded diagnostic modelling based on existing observation and a data-adaptive machine learning approach), we find a contrasting response of WUE to drought between arid (WUE increases with drought) and semi-arid/sub-humid ecosystems (WUE decreases with drought), which is attributed to different sensitivities of ecosystem processes to changes in hydro-climatic conditions. WUE variability in arid ecosystems is primarily controlled by physical processes (i.e., evaporation), whereas WUE variability in semi-arid/sub-humid regions is mostly regulated by biological processes (i.e., assimilation). We also find that shifts in hydro-climatic conditions over years would intensify the drought effect on WUE. Our findings suggest that future drought events, when coupled with an increase in climate variability, will bring further threats to semi-arid/sub-humid ecosystems and potentially result in biome reorganization, starting with low-productivity and high water-sensitivity grassland.

Ecosystem water use efficiency (WUE), herein defined as the ratio of carbon gain (i.e., Gross Primary Production, GPP) to water consumption (i.e., Evapotranspiration, ET), links the coupled carbon and water cycles and is a key variable to understand the response of ecosystem productivity to water availability^1^,^2^. Additionally, it also highlights the linkage between biological processes (i.e., photosynthesis and transpiration) and physical process (i.e., evaporation) that govern the Earth’s carbon and water budgets. On the other hand, the strong coupling of the carbon and water cycles implies that any environmental disturbance on one component of WUE (i.e., GPP or ET) simultaneously impacts the other. For example, drought is an intermittent disturbance of the water cycle that also heavily affects the terrestrial carbon cycle^3^,^4^,^5^. Historical records and model predictions suggest that there is an increasing trend of drought events, in terms of both frequency and intensity, which will have a profound impact on the coupled carbon and water cycles^5^,^6^,^7^. Hence, a better insight into the relationship between WUE and drought would greatly benefit our understanding of ecosystem processes, services and feedbacks to the climate system in the context of global change.

Numerous evidence has shown that, in most cases, drought suppresses both ecosystem productivity and ET simultaneously^3^,^4^,^5^,^7^,^8^,^9^,^10^. However, their magnitudes (or relative magnitudes), representing the sensitivity of different biological and/or physical processes to drought, depend on biome types and other confounding environmental factors, and varies considerably among studies^8^,^9^,^10^. As a result, a consensus of drought impacts on WUE has not yet been reached across all main global ecosystems. Here, we present the first global synthesis of the impact of drought on WUE by assessing the biome and ecosystem WUE responses to drought using two observation-based WUE datasets. First, observations of GPP and ET from 95 eddy covariance (EC) sites (526 site-years) were compiled to calculate WUE and examine the drought effect at the site level^11^. The observations cover a broad range of bio-climates and each site has at least three-years continuous measurements. Second, global monthly 0.5° gridded WUE estimates for 1982–2011 were determined from monthly GPP and ET estimates based on a data-adaptive machine learning approach (i.e., model tree ensembles, MTE) trained with FLUXNET data collected globally^12^. Compared to process-oriented ecosystem models, results of MTE are much less contingent on theoretical-model assumptions and are considered an independent data benchmark for numerous ecosystem models^9^,^10^,^12^. In addition, two drought indicators were used in this analysis, including the wetness index (WI = precipitation/potential ET) that quantifies the meteorological drought^13^ and the Palmer Drought Severity Index (PDSI)^14^ which quantifies hydrological drought by accounting for soil water storage dynamics (see Methods).

## Results and Discussion

Global mean annual WUE exhibits a remarkable spatial heterogeneity (see Supplementary Fig. S1), spanning a range from less than 0.5 g C/kg H_2_O in arid regions (i.e., North Africa, Central Euro-Asia and the western coast of South America) to higher than 4.0 g C/kg H_2_O in more humid areas (i.e., Western Europe). WUE and vegetation cover (as indicated by satellite-based Normalized Difference Vegetation Index, NDVI) is highly consistent, with higher NDVIs generally correspond to higher WUEs (*R*^2^ = 0.411, *P* < 0.001) (see Supplementary Fig. S1), supporting the results from previous studies in Europe^15^, North America^16^ and China^17^. At the biome level, forest ecosystems show higher WUEs than non-forest ecosystems, whereas the WUE spatial variability is lower in high WUE regions than that in low WUE regions (see Supplementary Fig. S2).

**Figure 1:**
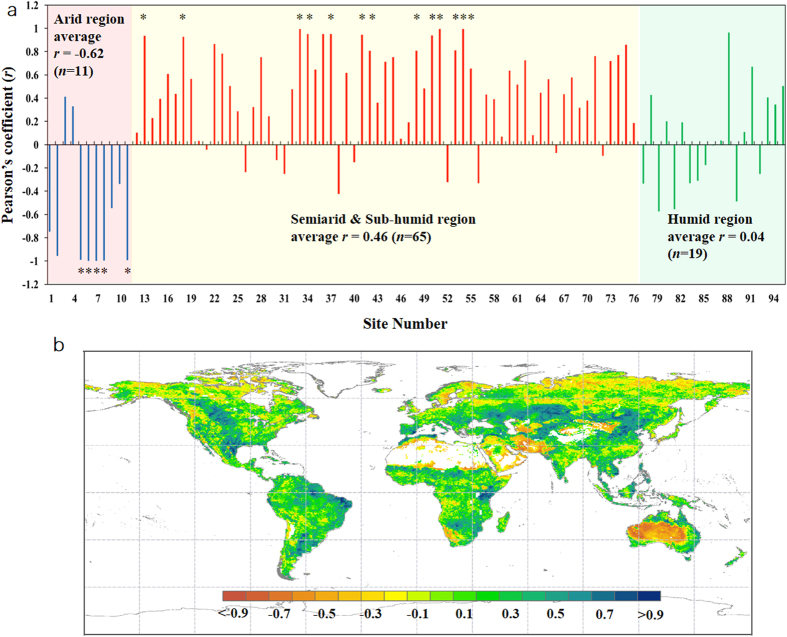
Effect of current year drought on ecosystem WUE at the site and grid-box levels. (**a**) Pearson’s coefficient between annual WUE and wetness index for each flux site. (*) indicates the regression is significant at the significance level of 90% (not at 95% due to a limited number of years at each site), and (**b**) Spatial distribution of Pearson’s coefficient (*r*) between detrended annual MTE-WUE and detrended annual PDSI series. Dashed grey lines are separated by 30° latitude or longitude from the equator and 0° E (as in all maps). Map was created using ArcMap 10.2 (http://www.esri.com/software/arcgis/arcgis-for-desktop).

**Figure 2:**
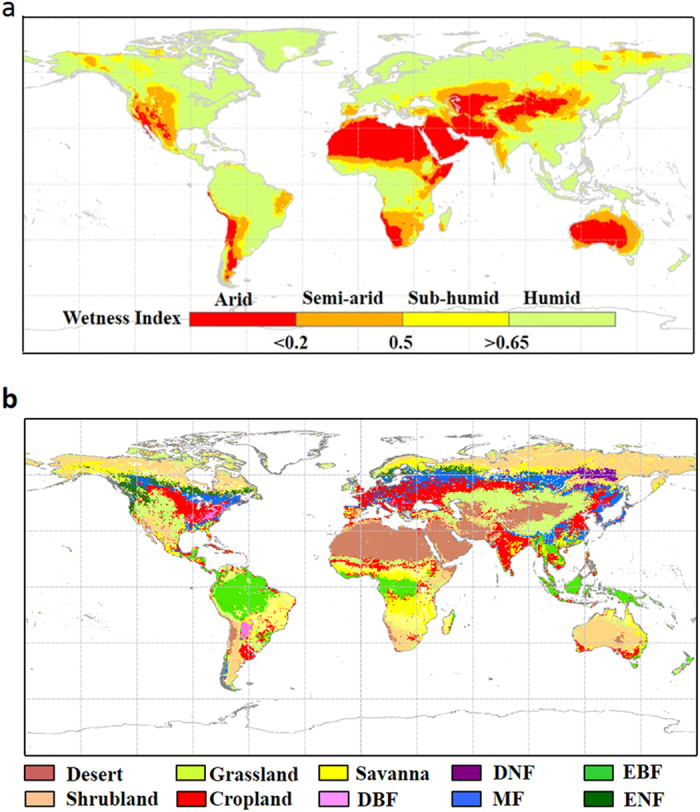
Global distribution of (**a**) mean annual wetness index zones for 1982–2011 and (**b**) biome types. The wetness index is calculated as the ratio of annual precipitation over annual Penman potential evapotranspiration, and the global wetness index zones are determined according to UNEP aridity classification^13^. Biome types include desert, shrubland, grassland, cropland, savanna, deciduous broadleaf forest (DBF), deciduous needle-leaf forest (DEF), mixed forest(MF), evergreen needle-lead forest (NEF) and evergreen broadleaf forest (DBF) are determined based on MODIS UMD 1-km land cover product (MOD12Q1). Maps were drawn using ArcMap 10.2 (http://www.esri.com/software/arcgis/arcgis-for-desktop).

To examine the drought-WUE relationship, all quantities are firstly detrended (by subtracting the linear trend derived from the least-square regression from the original data series– see Methods for more details) to focus the correlation analysis on interannual variability. Both WUE datasets reveal a contrasting response of WUE to drought across global ecosystems (Fig. 1), which corresponds well with global climatic zones (Fig. 2a). At the EC site level, nine of eleven (i.e., ~82%) arid sites show a negative relationship between WUE and WI, with *p* < 0.1 at five of them (*F*-test). In contrast, among the 65 examined EC sites in the semi-arid/sub-humid region, 50 of them (i.e., ~77%) show a positive relationship between WUE and WI (*p* < 0.1 at 14 sites). For the remaining 19 EC sites located in the humid zone, the WUE-WI relationship is positive in 11 sites and negative in 8 sites (*p* > 0.1 at all 19 humid sites).

Similar results were obtained from the correlation analysis between MTE-WUE and PDSI (Fig. 1b). Significant negative correlations (i.e., *p* < 0.05) between WUE and PDSI are observed in arid areas, including central and western Asia, southern Africa and the majority of central and western Australia. In contrast, positive relationships between WUE and PDSI occur in semi-arid and sub-humid regions, i.e., the Great Plain of North America, a large portion of central Euro-Asia, the south-eastern Amazon Basin, the western part of the Pampas Steppe (also in South America) and areas surrounding the Congo Basin in Africa. For humid regions, no significant relationship between WUE and PDSI is observed (*p* < 0.05), suggesting that drought has limited effects on ecosystem WUE in non-water limited regions.

**Figure 3:**
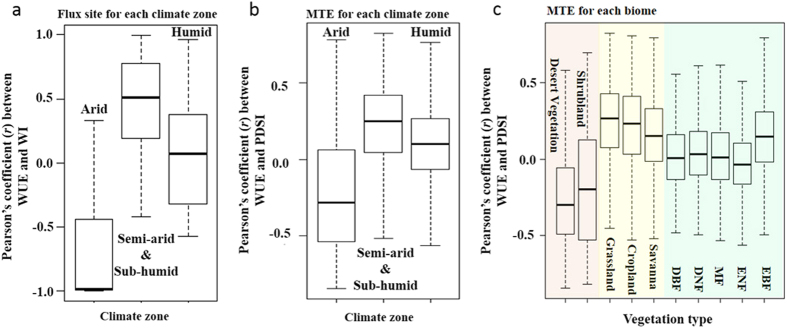
Effect of current-year drought on ecosystem WUE at the climate zone and biome levels. Boxplot of Pearson’s coefficient between (**a**) detrended annual Flux site-WUE and WI for each climate zone, (**b**) detrended annual MTE-WUE and PDSI for each climate zone, and (**c**) detrended annual MTE-WUE and PDSI for each vegetation type. The background colours in (**c**) indicate different climate zones (red: arid zone; yellow: semi-arid/sub-humid zone; green: humid zone and the five vegetation types for the humid zone are expanded in Fig. 2). Each box represents the inter-quartile range of the data (25%~75% quantile), the dark horizontal line within the box indicates the median and the whiskers (i.e., highest and lowest horizontal lines connected to the box by dashed lines) are the maximum and minimum data values, respectively. Note that the boxplot analysis was not undertaken for Pearson’s coefficient between Flux site-WUE and WI at the biome level due to limited flux sites within each biome. Images were drawn by using R 3.1.2 (https://www.r-project.org/).

The distinct response of WUE to drought between arid and semi-arid/sub-humid regions is reinforced when averaging the Pearson’s coefficient between WUE and WI (or PDSI) by biome types (Fig. 3c) and/or climate zones (Fig. 3a,b). The good agreement between climate zone and vegetation functional type demonstrates a predominant role of climate in shaping ecosystems^18^. Consistent with the results shown in Fig. 1, arid zone WUE (i.e., desert vegetation and shrubland) responds negatively with an increase in WI (or PDSI), with the negative correlation becoming more significant for areas of a drier climate (Fig. 3). In contrast, the WUE and WI (or PDSI) relationship is completely reversed to a positive relationship for biomes in semi-arid and sub-humid regions (i.e., grassland, croplands and savanna, Fig. 3). Interestingly, this positive relationship becomes weaker for areas of increased climate wetness and disappears in humid zones except for evergreen broad leaved forest located in the eastern Amazon Basin (Figs 1 and 3). Similar results are observed when the analyses are performed based on non-detrended and/or using WI instead of PDSI in the MTE-WUE data (see Supplementary Figs S3–S5). The fact that using the non-detrended time series results in the same conclusion (as using detrended variables) suggests that the long-term trends in both WUE and drought has not broken the interannual correlations between the two variables.

**Figure 4:**
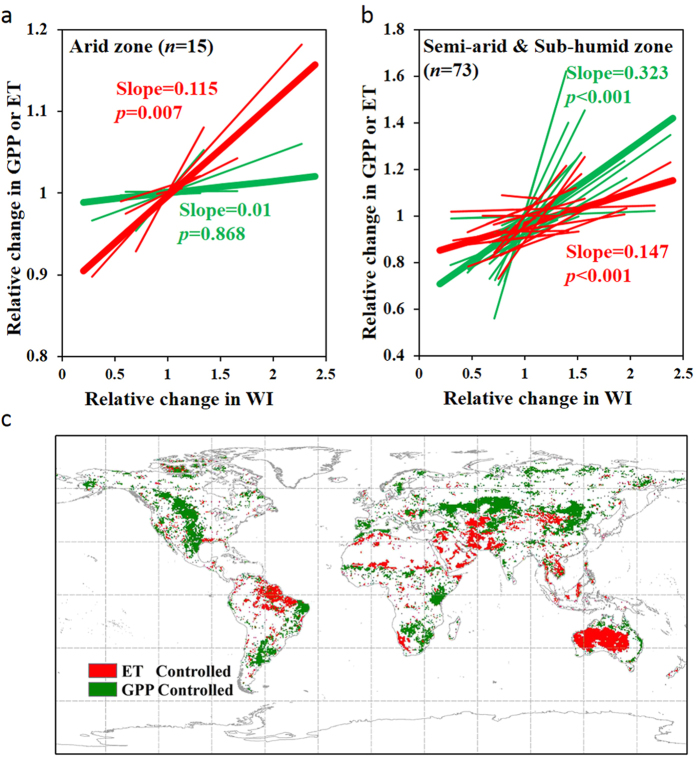
Relative sensitivity of GPP and ET to drought. (**a**) linear regression lines between normalized GPP (or ET) and normalized WI for each flux site and across sites within the arid zone and (**b**) within the semi-arid and sub-humid zones; (**c**) spatial distribution of the dominant controlling factor (i.e., GPP or ET) of annual 1982–2011 WUE variability in response to drought using the MTE data. In (**a**,**b**), only sites with the correlation between WUE and WI being significant (*p* < 0.1) are shown. Fine lines indicate the best linear regression at each flux site and bold lines represent the linear regression pooled across all significant sites. Slope and *p*-value are reported for bold lines. In (**c**), only pixels with correlations in Fig. 1b being significant (*p* < 0.05) are shown. Map was created using ArcMap 10.2 (http://www.esri.com/software/arcgis/arcgis-for-desktop).

As an integrated measure of the coupled water and carbon cycles, ecosystem WUE is determined by multiple, and often interacting physical and biological processes. The contrasting response of WUE to drought between arid and semi-arid/sub-humid biomes suggests a different sensitivity of ecosystem processes to drought among those biomes. Vegetation in arid areas are likely to have already adapted to the dry climate and hence possesses a generally greater tolerance to drought^19^,^20^. Consequently, plants would still perform relatively well when drought persists, implying a relatively low sensitivity of GPP to drought in arid ecosystems (Fig. 4a,c and Supplementary Figs S6 and S7). On the other hand, additional water input in wet years cannot be effectively used by plants due to low vegetation coverage, resulting in higher percentage of non-biological water consumption (i.e., soil evaporation) in arid regions^21^. Therefore, the higher ET sensitivity to changes in hydro-climatic conditions is the primary reason for the negative correlation between WUE and WI (or PDSI) in arid ecosystems (although the overall sensitivity of GPP and ET to drought is low in arid ecosystems). In contrast, ecosystems in semi-arid and sub-humid regions are dominated by herbaceous plants (i.e., grassland, cropland and savanna) whose functions and activities depend largely on water availability^22^,^23^,^24^. Studies have shown that ecosystem productivity in grassland responds most rapidly to precipitation variability^22^,^24^, suggesting a high GPP sensitivity to water availability in these climate zones. In semi-arid and sub-humid regions, deeper rooted perennial vegetation also responds to precipitation variability, albeit on a longer time-scale than grasses, as has been observed using satellite remote sensing^25^,^26^ and in numerous field studies^27^,^28^,^29^,^30^,^31^. In these semi-arid and sub-humid ecosystems, although ET decreases simultaneously during drought, its response is smaller than the GPP response, leading to a lower WUE in drier years and vice versa (Fig. 4b,c and Supplementary Figs S6 and S7). The above analyses are supported by comparing the relative sensitivities of GPP and ET to WI (and/or PDSI) in both datasets (Fig. 4), which shows that ET dominates WUE variability in arid ecosystems, whereas GPP dominates WUE variability in semi-arid and sub-humid regions.

Our analysis also reveals an ET-dominated WUE variability in tropical humid regions (i.e., Amazon, Congo and southeast Asia) (Fig. 4c), where variation of ecosystem functions are largely driven by energy supply rather than water availability^32^,^33^. Drought is often caused by less precipitation (and thus less cloud cover), which results in more incoming solar radiation and therefore accelerates ecosystem processes (e.g., GPP and ET)^33^. On the other hand, studies have shown that nutrient availability plays an important role in determining vegetation growth in the absence of water and temperature stresses^25^, but nutrient availability is less likely to directly affect the physical processes of ET (i.e., soil evaporation and evaporation from canopy interception). This may explain the overall lower sensitivity of GPP to drought than the sensitivity of ET to drought in these tropical rainforest regions.

**Figure 5:**
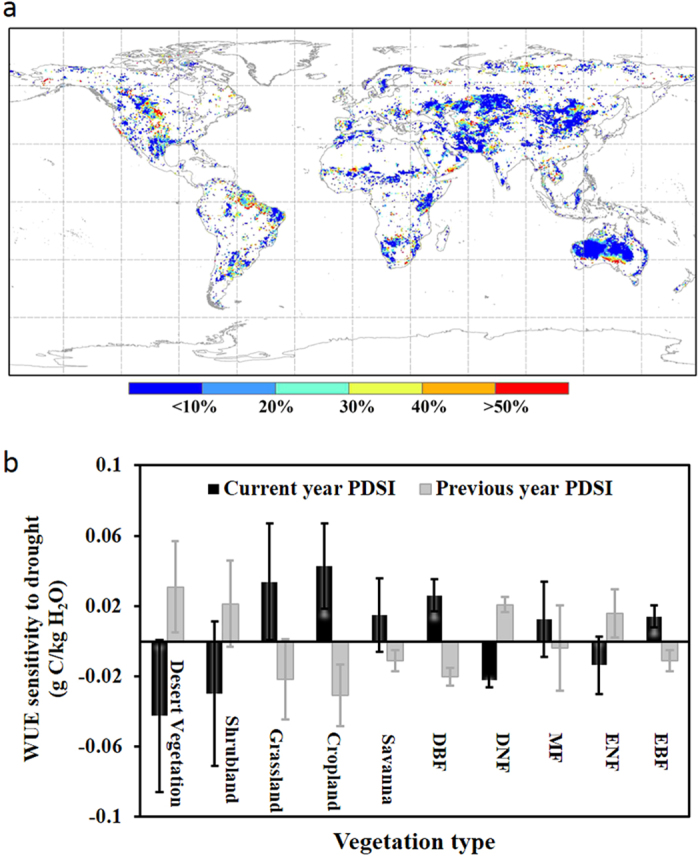
Memory effect of previous-year drought on WUE. (**a**) The independent effect of previous-year drought on the goodness of fit between annual detrended MTE-WUE and PDSI and (**b**) WUE sensitivity to the current-year PDSI and previous-years PDSI. This analysis was only performed on the annual gridded 1982–2011 MTE data due to the relative short length of the EC flux site data. Map was created using ArcMap 10.2 (http://www.esri.com/software/arcgis/arcgis-for-desktop).

Many studies have suggested that drought legacy affects ecosystem productivity and water consumption^36^,^37^,^38^,^39^: known as “the memory effect”. Here we show that the memory effect of previous-year drought on current-year WUE is higher in three regions, i.e., the Great Plain, the Amazon Basin and the part of Australia experiencing a Mediterranean climate, but generally low in other areas (Fig. 5a, by conducting the hierarchical partitioning analysis^40^). Further support is given by the Akaike Information Criterion analysis^41^ on the WUE model by current-year PDSI and by two-year PDSIs (see Supplementary Fig. S8). Although the effect of hydro-climatic disturbance on WUE varies greatly among bio-climates, the memory effect shows a pattern where the impact of previous-year drought consistently shows an opposite sign than that of the current-year drought on WUE for all biomes (Fig. 5b). This result suggests that the memory effect enhances (weakens) the impact of current-year hydro-climatic disturbance on WUE if there is (not) a transition in the disturbance between the two consecutive years. Transitions in hydro-climatic conditions bring further disturbance into the ecosystem, which will lead to greater variability in ecosystem functions and activities. In contrast, a stable hydro-climatic condition between years allows an ecosystem to better adapt to the environment and thus they are less responsive to the current hydro-climatic condition. This finding also provides evidence of ecosystem acclimation at a relatively short time scale (i.e., annual).

Our findings have important implications for studying the effect of climate change on ecosystem behaviour. The contrasting response of ecosystem WUE to drought, which results from different sensitivity of ecosystem processes to changes in hydro-climatic conditions, implies that biological (i.e., GPP) controlled ecosystems (i.e., semi-arid/sub-humid ecosystems) may be more vulnerable to drought than physical (i.e., evaporation) controlled ecosystems (i.e., arid ecosystems), confirming previous findings^22^,^23^,^24^. On the other hand, climate change is predicted to increase both the frequency and intensity of drought^6^, and when coupled with continued and substantial warming, a new hydro-climatological regime will likely form in many areas^6^. This is especially important for semi-arid/sub-humid ecosystems, as more severe droughts could result in a larger reduction in their WUE by reducing ecosystem productivity or considerable vegetation mortality^5^. If drought continues, breakdown in ecosystem resilience may occur as ecosystem WUE reduces to a certain threshold, which, when crossed, can result in biome reorganization^24^. This phenomenon has already been observed during large-scale shrub encroachment in North America^42^ and grassland desertification in Central Euro-Asia^43^. Moreover, such processes may be further accelerated by the memory effect of drought on ecosystem processes if climate variability increases concurrently with droughts.

## Methods

### Data sets

Our analyses are based on two WUE data sets that are both ultimately derived from EC measurements. Firstly, GPP and ET observations at 95 flux sites (526 site-years) were obtained (Supplementary Table S1). At each site, H_2_O flux, CO_2_ flux and meteorological variables were measured at 30-minute intervals using the EC technique. Half-hourly GPP was calculated during daylight hours as net ecosystem exchange minus ecosystem respiration using the standard FLUXNET on-line flux partitioning tool^44^. Half-hourly data were accumulated to obtain GPP and ET daily, monthly and annually. All sites have at least three years continuous measurements and years with the average EC data quantity score larger than 0.8 (on a scale of 0–1)^11^. These sites cover a broad range of vegetation types and climate zones, representing a “global context”.

The second global GPP and ET dataset were produced by upscaling FLUXNET observations of CO_2_ and H_2_O fluxes to the global scale using a data-adaptive machine learning technique (i.e., model tree ensembles (MTE))^12^. The MTE model was first trained at site level and then applied to generate monthly global flux fields at a 0.5° spatial resolution from 1982 to 2011. A ten-fold cross validation was performed to evaluate the MTE models, and the results show a high accuracy of predicated GPP and ET by the MTE models, with *R*^2^ between observed and estimated measures being 0.85 (n = 4209) for GPP^9^ and 0.91 (n = 4678) for ET^10^.

Drought was also quantified by two different drought indicators, i.e., WI and PDSI. Monthly global PDSI at a 0.5° spatial resolution from 1979 to 2011 was calculated following the method in ref. 3, except that the potential evapotranspiration (PET) is calculated using the fully-physically based Penman Equation^45^. PDSI from 1982 to 2011 was used to quantify drought (i.e., the lower the PDSI value the more severe the drought is). However, due to relative short observation period, PDSI was not calculated at the site level. Instead, WI was used to quantify drought for each EC site. Globally, WI was calculated using monthly meteorological data (0.5° spatial resolution) obtained from the Climate Research Unit (CRU TS 3.2)^46^, and at the site level using meteorological data observed directly by the flux tower. The global aridity zones were defined based on WI following the UNEP aridity classification^13^.

Land cover types include desert, shrubland, grassland, cropland, savanna, deciduous broadleaf forest (DBF), deciduous needle-leaf forest (DNF), mixed forest (MF), evergreen needle-leaf forest (ENF) and evergreen broadleaf forest (EBF) were classified based on the MODIS UMD 1-km land cover product (MOD12Q1)^47^.

### Analysis

To focus the analysis on interannual variability, all variables (both WUE datasets and the two drought indexes) were detrended by subtracting the linear trend derived from the least-square regression from the original data series. Such treatment also minimizes the effects of other monotonic environmental factors on long-term WUE trend (e.g., elevation in atmospheric CO_2_ concentration^48^). Then, a liner correlation analysis was performed between the detrended annual WUE and drought indexes to investigate the drought effect on interannual variability in WUE. The same analyses were performed with non-detrended data, resulting in similar conclusions (see Supplementary Figs S5–S7).

To determine the dominant factor (i.e., GPP or ET) that controls the interannual variability of WUE in response to drought, the relative sensitivity of GPP (or ET) to drought were quantified as the slope of the linear regression function between normalized GPP (or ET) and normalized drought index (each normalized by the mean value through the time series). If the relative sensitivity (i.e., the slope of the linear regression) of GPP to drought is higher than that of ET to drought, variability in WUE at the corresponding site (or pixel) is considered to be GPP-controlled, otherwise it is ET-controlled.

To examine the memory effect of previous year drought on current-year WUE, we built a dualistic linear regression models between current-year WUE (i.e., WUE_current_ = *a* × PDSI_current_ + *b*) and previous-year and current-year PDSI (i.e., WUE_current_ = *a* × PDSI_current_ + *b* × PDSI_previous_ + *c*). Due to the short data length at the EC flux sites, analysis of the memory effect was only performed annually using the gridded MTE-WUE data from 1982 to 2011. The hierarchical partitioning algorithm^40^ was used to determine the independent effects of current-year and previous-year PDSI on WUE as a percentage contribution to the goodness of fit of the dualistic linear regression model. The Akaike Information Criterion (AIC) analysis^41^ was also performed to evaluate the benefit of adding the previous-year PDSI to the regression model between PDSI and WUE (see Supplementary Fig. S10). The new model (using two years PDSI) is considered as an improvement over the old one (using one year PDSI) if the AIC value reduced by more than 2.0.

## Additional Information

**How to cite this article**: Yang, Y. *et al.* Contrasting responses of water use efficiency to drought across global terrestrial ecosystems. *Sci. Rep.* **6**, 23284; doi: 10.1038/srep23284 (2016).

## Supplementary Material

Supplementary Information
