# Supplementary material for: Contrasting responses of water use efficiency to drought across global terrestrial ecosystems
Source: Sci Rep. 2016 Mar 17;6:23284. doi: 10.1038/srep23284 (PMC4794702; doi:10.1038/srep23284)
Supplement: Supplementary Information [file srep23284-s1.pdf]

Supplementary Information

Contrasting responses of water use efficiency to drought across global terrestrial ecosystems

Yuting Yang[1,2,*], Huade Guan[1,3], Okke Batelaan[1,3], Tim R. McVicar[2,4], Shilong Piao[5], Di Long[6], Wei Liang[7], Bing Liu[8], Zhao Jin[7], Craig Simmons[1,3]

[1]School of the Environment, Flinders University, Adelaide, South Australia 5042, Australia

[2]CSIRO Land and Water, Canberra, ACT 2601, Australia

[3]National Centre for Groundwater Research and Training, Adelaide, South Australia 5042, Australia

[4]Australian Research Council Centre of Excellence for Climate System Science, Sydney, Australia

[5]Sino-French Institute for Earth System Science, College of Urban and Environmental Sciences, Peking University, Beijing 100871, China.

[6]State Key Laboratory of Hydroscience and Engineering, Department of Hydraulic Engineering, Tsinghua University, Beijing 100084, China

[7]College of Tourism and Environmental Sciences, Shaanxi Normal University, Xi'an 710062, China

[8]Linze Inland River Basin Research Station, Laboratory of Heihe River Eco-Hydrology and Basin Science, Cold and Arid Regions Environmental and Engineering Research Institute, Chinese Academy of Sciences, Lanzhou 730000, China

*Author to whom correspondence should be addressed; Email: yuting.yang@csiro.au; Tel.: +61-2-62183448;

(Surnames are underlined)

This supplementary material file includes:

Supplementary Figures S1 to S8

Supplementary Tables S1

Section 1. Global spatial patterns of annual WUE and NDVI.

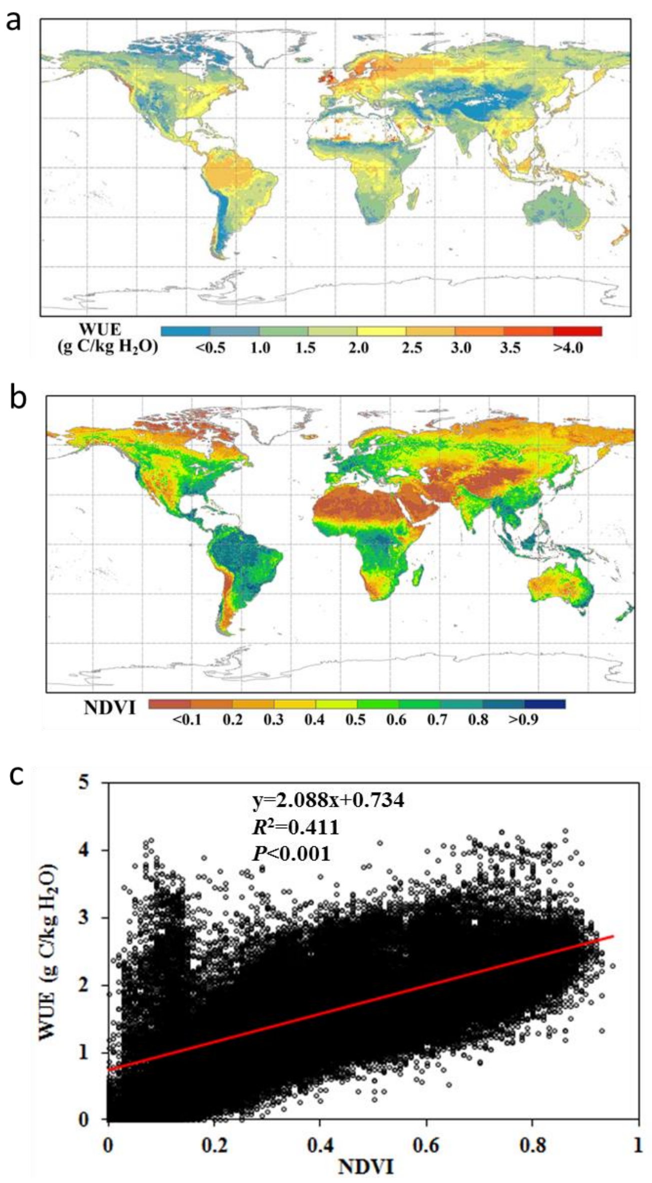

Supplementary Figure S1 Global distribution of mean annual WUE and NDVI over 1982-2011. (a) global distribution of mean annual WUE calculated over 1982-2011 based on the MTE data, (b) global distribution of mean annual NDVI over 1982-2011 calculated based on the AVHRR-GIMMS-3g NDVI dataset, and (c) relationship between mean annual WUE and NDVI over 1982-2011. The red solid line in (c) indicates the best linear fit. Note there are no MTE data over Sahara, Greenland and Antarctica regions. Map was drawn using ArcMap 10.2.

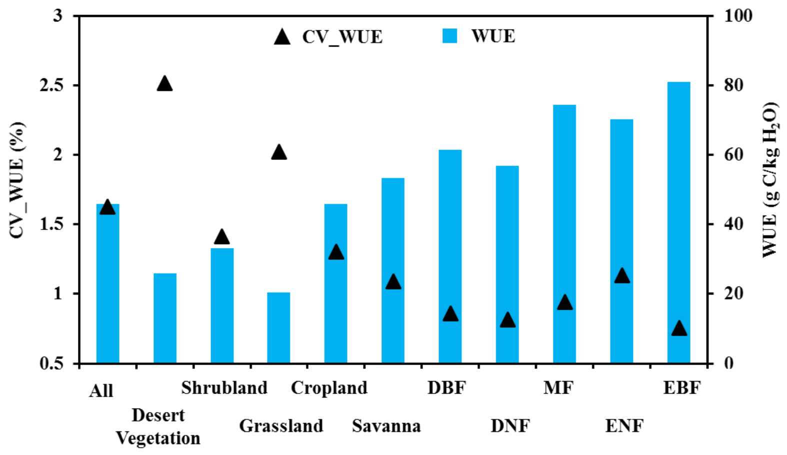

Supplementary Figure S2 Mean (1982-2011) and spatial variability of mean annual WUE for each vegetation type based on the MTE data. The spatial variability of the entire biome class is indicated by the coefficient of variation (CV). The results show that higher WUEs are generally found in forest ecosystems, whereas the spatial variability of WUE is lower in high WUE regions than in low WUE regions. Image was drawn using Excel 2013.

Section 2. Drought effect on the WUE

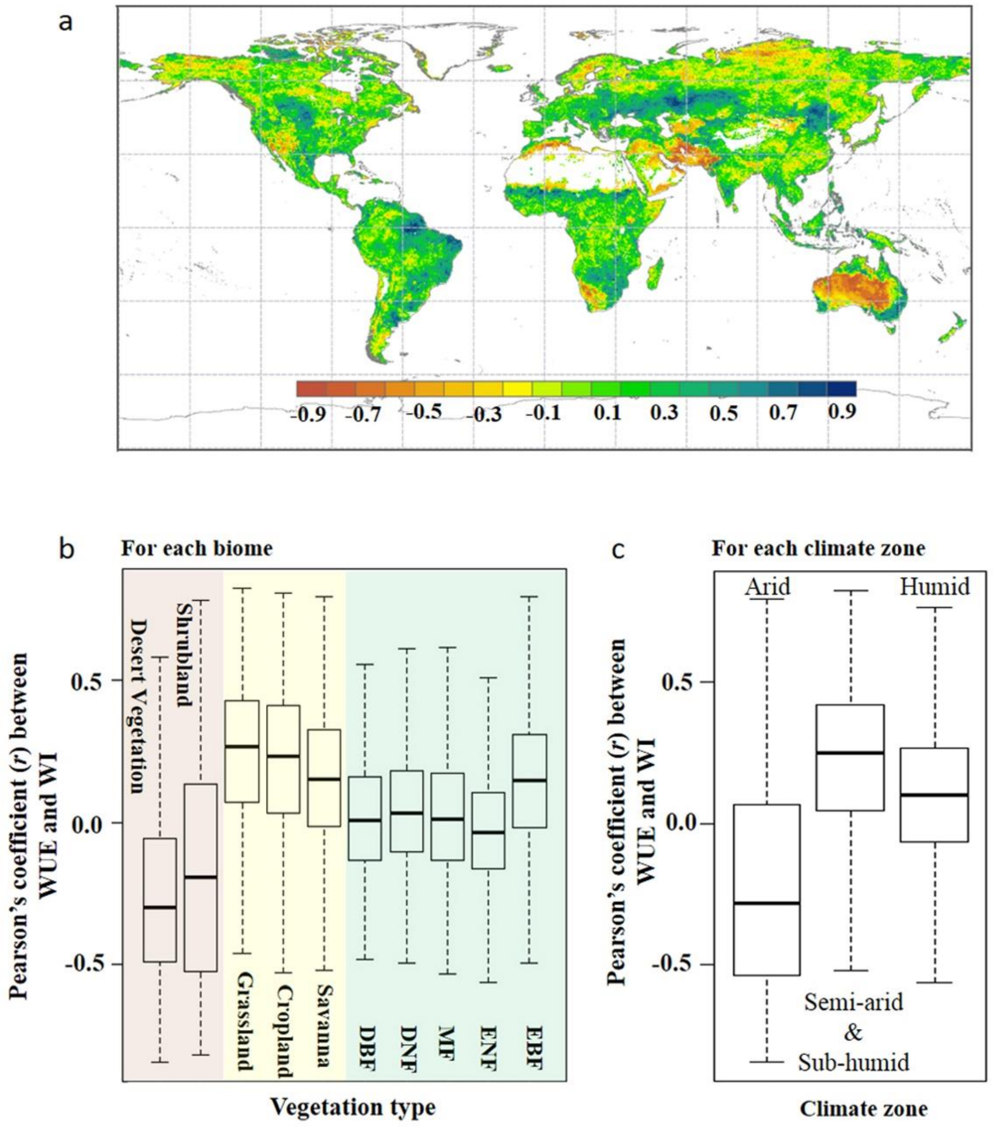

Supplementary Figure S3 Relationship between non-detrended annual MTE-WUE and PDSI series over 1982-2011. (a) Spatial distribution of Pearson's coefficient (*r*) between non-detrended annual MTE-WUE and PDSI series, (b) boxplot of Pearson's coefficient between non-detrended annual MTE-WUE and PDSI for each biome, (c) boxplot of Pearson's coefficient between non-detrended annual MTE-WUE and PDSI for each climate zone. The background colours in (b) indicate different climate zones (red: arid zone; yellow: semi-arid/sub-humid zone; green: humid zone). The interpretation of the boxplots is given in Figure 2. Map was drawn using ArcMap 10.2 and boxplots were drawn using R 3.1.2.

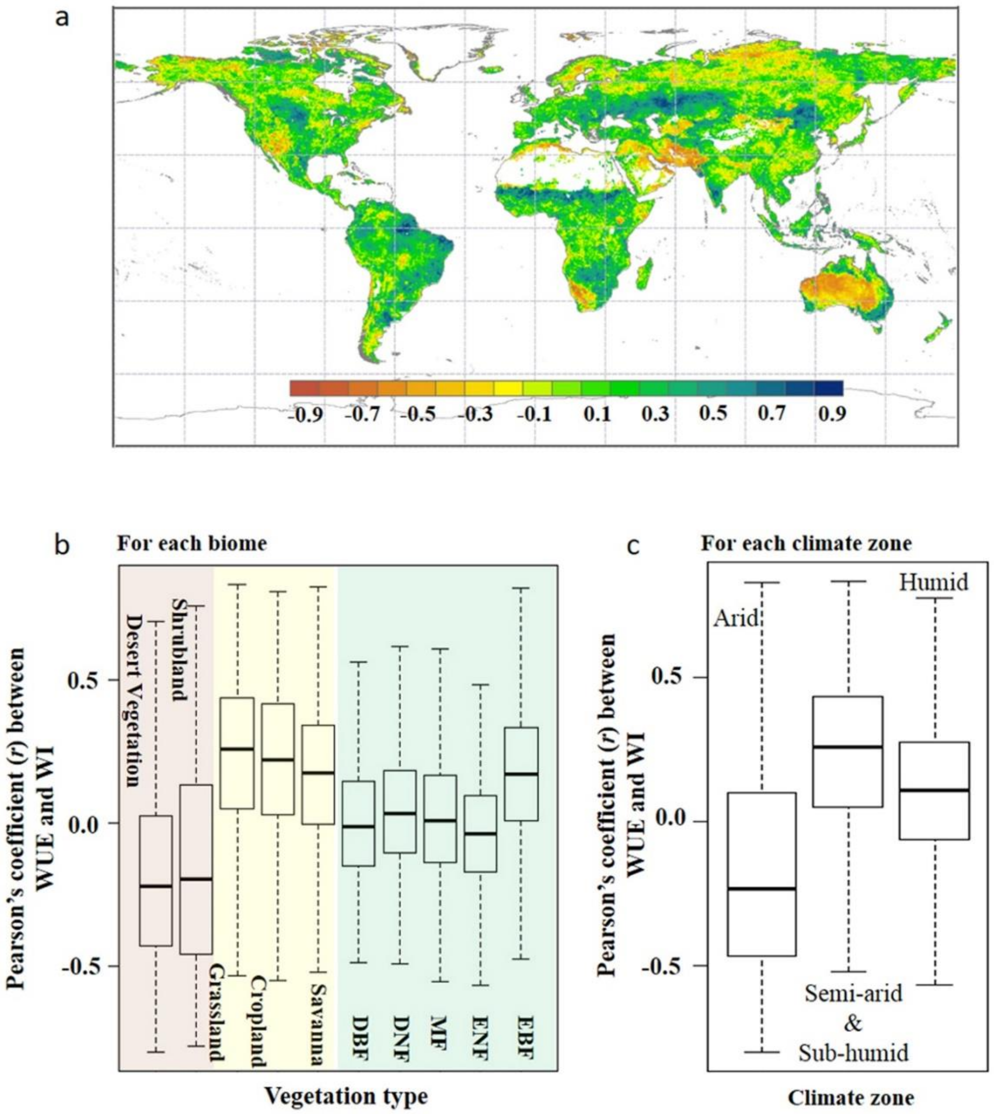

Supplementary Figure S4 Relationship between detrended annual MTE-WUE and WI series over 1982-2011. (a) Spatial distribution of Pearson's coefficient (*r*) between detrended annual MTE-WUE and WI series, (b) boxplot of Pearson's coefficient between detrended annual MTE-WUE and WI for each biome, (c) boxplot of Pearson's coefficient between detrended annual MTE-WUE and AI for each climate zone. The background colours in (b) indicate different climate zones (red: arid zone; yellow: semi-arid/sub-humid zone; green: humid zone). The interpretation of the boxplots is given in Figure 2. Map was drawn using ArcMap 10.2 and boxplots were drawn using R 3.1.2.

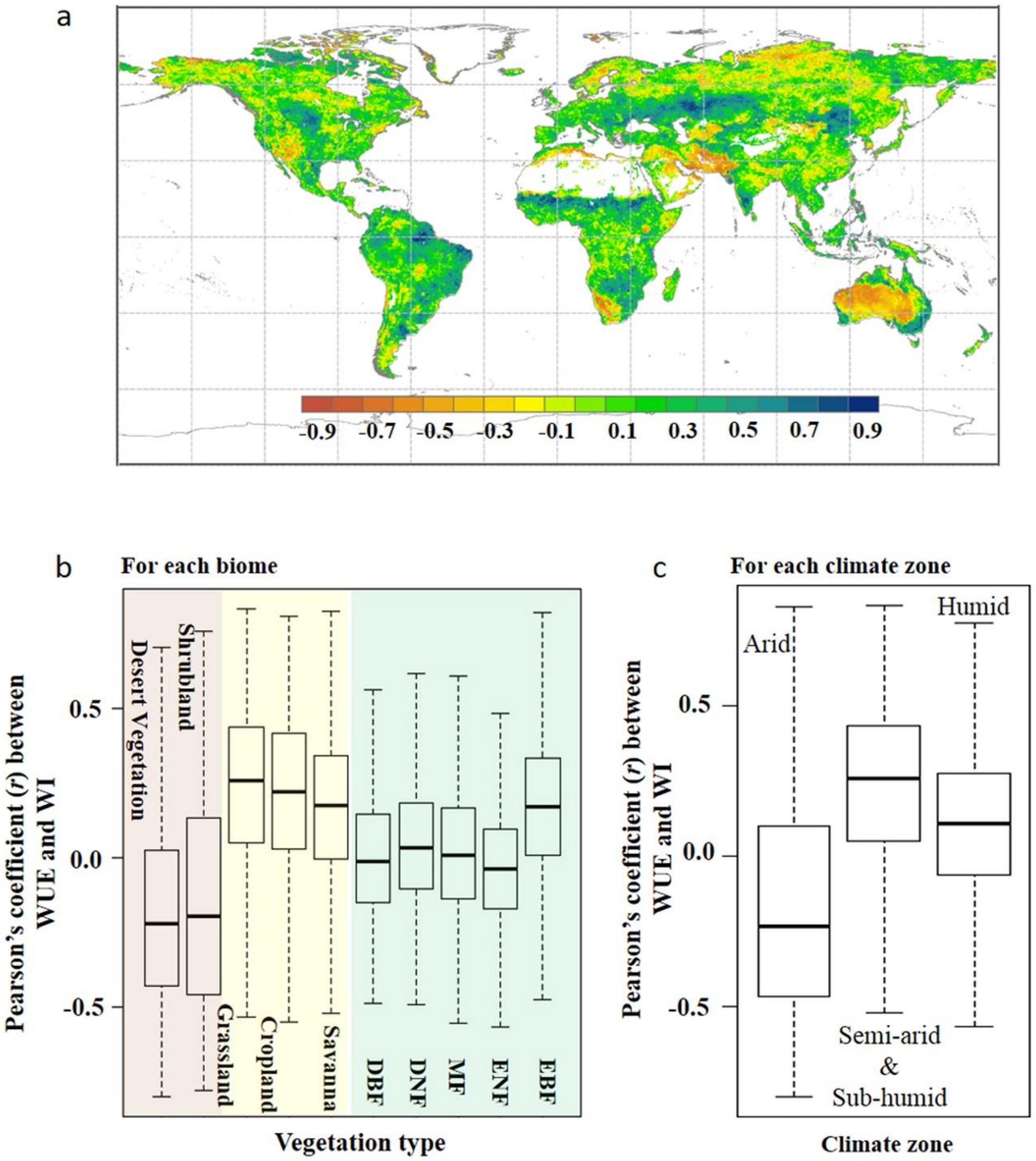

Supplementary Figure S5 Relationship between non-detrended annual MTE-WUE and WI series over 1982-2011. (a) Spatial distribution of Pearson's coefficient (*r*) between non-detrended annual MTE-WUE and WI series, (b) boxplot of Pearson's coefficient between non-detrended annual MTE-WUE and WI for each biome, (c) boxplot of Pearson's coefficient between non-detrended annual MTE-WUE and WI for each climate zone. The background colours in (b) indicate different climate zones (red: arid zone; yellow: semi-arid/sub-humid zone; green: humid zone). The interpretation of the boxplots is given in Figure 2. Map was drawn using ArcMap 10.2 and boxplots were drawn using R 3.1.2.

Supplementary Table S1 Descriptions of the flux sites used in this study including site number, site identifier (Site ID), latitude (Lat), Longitude (Lon), mean wetness index during the data period (Mean WI), climate zone (based on Mean WI), correlation coefficient between annual WUE and WI (*r*) and the *p*-value, and references (Ref).

Site Number	Site ID	Lat (ºN)	Lon (ºE)	Biome Type	Mean WI	Climate Zone	*r*	*p*-value	Ref
1	BW-Ma1	-19.92	23.56	Savanna	0.07	Arid	-0.748	0.462	2
2	CA-NS1	55.88	-98.48	Shrub	0.11	Arid	-0.955	0.191	3
3	CA-NS3	55.91	-98.38	ENF	0.12	Arid	0.413	0.489	3
4	CA-NS6	55.92	-98.96	Shrub	0.16	Arid	0.329	0.671	3
5	CA-NS7	56.64	-99.95	Shrub	0.16	Arid	-0.989	0.096	4
6	CA-SF3	54.09	-106.00	Shrub	0.11	Arid	-0.999	0.027	5
7	ES_Lma	39.94	-5.77	Shrub	0.18	Arid	-0.998	0.037	6
8	US-FR2	29.95	-98.00	Savanna	0.12	Arid	-0.995	0.064	7
9	US-SRM	31.82	-110.87	Savanna	0.09	Arid	-0.546	0.642	8
10	US-Wkg	31.74	-109.94	Grass	0.07	Arid	-0.337	0.781	9
11	US_Me5	44.44	-121.57	Savanna	0.15	Arid	-0.994	0.07	10
12	AU-Tum	-35.66	148.15	EBF	0.57	Sub-humid	0.104	0.868	11
13	AU-How	-12.49	131.15	Savanna	0.43	Semi-arid	0.937	0.019	12
14	BE_Vie	50.31	6.00	MF	0.54	Sub-humid	0.228	0.556	13
15	CA-Man	55.88	-98.48	ENF	0.21	Semi-arid	0.394	0.44	14
16	CA-Mer	45.41	-75.52	Wetland	0.50	Sub-humid	0.608	0.184	15
17	CA-NS2	55.91	-98.52	ENF	0.21	Semi-arid	0.436	0.566	3
18	CA-NS5	55.86	-98.49	ENF	0.22	Semi-arid	0.928	0.0721	3
19	CA-Qfo	49.69	-74.34	ENF	0.64	Sub-humid	0.567	0.433	16
20	CA-SF2	54.25	-105.88	ENF	0.21	Semi-arid	0.034	0.978	5
21	DE_Geb	51.10	10.91	Crop	0.58	Sub-humid	-0.043	0.973	17
22	DE_Hai	51.08	10.45	DBF	0.53	Sub-humid	0.866	0.012	18
23	DE_Kli	50.89	13.52	Crop	0.45	Semi-arid	0.784	0.426	19
24	DE-Tha	50.96	13.57	ENF	0.52	Sub-humid	0.504	0.138	20
25	ES_ES1	39.35	-0.32	ENF	0.22	Semi-arid	0.287	0.5	21
26	ES_ES2	39.28	-0.32	Crop	0.22	Semi-arid	-0.238	0.847	21
27	ES_VDA	42.15	1.45	Grass	0.58	Sub-humid	0.325	0.789	22
28	FI-Hyy	61.85	24.29	ENF	0.31	Semi-arid	0.754	0.031	23
29	FR-LBr	44.72	-0.77	ENF	0.38	Semi-arid	0.244	0.56	24
30	FR_Pue	43.74	3.60	EBF	0.40	Semi-arid	-0.132	0.777	25
31	FR-Hes	48.67	7.06	DBF	0.51	Sub-humid	-0.251	0.551	26
32	HU_Bug	46.69	19.60	Grass	0.28	Semi-arid	0.476	0.424	27
33	HU_Mat	47.85	19.73	Grass	0.31	Semi-arid	0.994	0.069	27
34	IL-Yat	31.34	35.05	ENF	0.25	Semi-arid	0.952	0.048	28
35	IT_LMa	45.58	7.15	Grass	0.27	Semi-arid	0.644	0.167	29
36	IT_PT1	45.20	9.06	DBF	0.32	Semi-arid	0.951	0.2	30
37	IT_Ren	46.59	11.43	ENF	0.49	Semi-arid	0.951	0.085	31
38	IT_Sro	43.73	10.28	ENF	0.28	Semi-arid	-0.425	0.401	32
39	IT-Amp	41.90	13.61	Grass	0.42	Semi-arid	0.619	0.266	22
40	IT-Cpz	41.71	12.38	EBF	0.31	Semi-arid	-0.152	0.7	33
41	IT-MBo	46.02	11.05	Grass	0.55	Sub-humid	0.944	0.056	34
42	IT-Ro1	42.41	11.93	DBF	0.28	Semi-arid	0.809	0.024	35
43	IT-Ro2	42.39	11.92	DBF	0.31	Semi-arid	0.362	0.764	36
44	NL-Ca1	51.97	4.93	Grass	0.45	Semi-arid	0.714	0.286	37
45	PT-Esp	38.64	-8.60	EBF	0.22	Semi-arid	0.751	0.459	38

46	RU-Fyo	56.46	32.92	ENF	0.41	Semi-arid	0.052	0.912	39
47	SE-Fla	64.11	19.46	ENF	0.33	Semi-arid	0.191	0.809	40
48	SE-Nor	60.09	17.48	ENF	0.40	Semi-arid	0.809	0.1	41
49	UK-Esa	55.91	-2.86	Crop	0.31	Semi-arid	0.482	0.68	42
50	UK-Gri	56.61	-3.80	ENF	0.57	Sub-humid	0.94	0.005	43
51	US-Wi4	46.74	-91.16	MF	0.24	Semi-arid	0.993	0.067	44
52	US-ARM	36.61	-97.49	Crop	0.22	Semi-arid	-0.322	0.688	45
53	US-Aud	31.59	-110.51	Grass	0.31	Semi-arid	0.81	0.1	46
54	US-Bkg	44.35	-96.84	Grass	0.37	Semi-arid	0.995	0.064	47
55	US-Blo	38.90	-120.63	ENF	0.35	Semi-arid	0.654	0.056	48
56	US-Bo1	40.01	-88.29	Crop	0.38	Semi-arid	-0.331	0.35	49
57	US-FPe	48.31	-105.10	Grass	0.38	Semi-arid	0.431	0.469	47
58	US-Ha1	42.54	-72.17	DBF	0.55	Sub-humid	0.392	0.233	50
59	US-Ho1	45.20	-68.74	ENF	0.43	Semi-arid	0.07	0.859	51
60	US-Me2	44.45	-121.56	ENF	0.25	Semi-arid	0.635	0.562	52
61	US-MMS	39.32	-86.41	DBF	0.39	Semi-arid	0.516	0.484	53
62	US-Ne1	41.17	-96.48	Crop	0.28	Semi-arid	0.726	0.276	54
63	US-Ne3	41.18	-96.44	Crop	0.24	Semi-arid	0.083	0.917	54
64	US-Ton	38.43	-120.97	Savanna	0.22	Semi-arid	0.446	0.376	55
65	US-UMB	45.56	-84.71	DBF	0.31	Semi-arid	0.564	0.322	56
66	US-Var	38.41	-120.95	Grass	0.50	Semi-arid	-0.072	0.892	55
67	US-WCr	45.81	-90.08	DBF	0.45	Semi-arid	0.433	0.271	57
68	US_Bar	44.06	-71.29	DBF	0.53	Sub-humid	0.578	0.607	58
69	US_IB2	41.81	-88.24	Crop	0.56	Sub-humid	0.319	0.681	59
70	US_NR1	40.03	-105.55	ENF	0.26	Semi-arid	0.38	0.312	60
71	US_SP3	29.75	-82.16	EBF	0.43	Semi-arid	0.763	0.11	61
72	US_SP1	29.74	-82.22	EBF	0.34	Semi-arid	-0.097	0.903	61
73	US_Ro1	44.71	-93.09	Crop	0.51	Sub-humid	0.718	0.49	62
74	US_Ro3	44.72	-93.09	Crop	0.46	Semi-arid	0.771	0.439	62
75	US_So4	33.37	-116.62	Savanna	0.36	Semi-arid	0.859	0.343	63
76	US-Syv	46.24	-89.35	MF	0.31	Semi-arid	0.187	0.813	64
77	AT-Neu	47.12	11.32	Grass	1.15	Humid	-0.335	0.665	65
78	BE_Bra	51.31	4.52	MF	0.66	Humid	0.427	0.291	66
79	CA-Qcu	49.27	-74.04	ENF	0.70	Humid	-0.573	0.313	16
80	CH-Oe1	47.29	7.73	Grass	0.78	Humid	0.202	0.798	67
81	DE_Meh	51.28	10.66	Grass	0.68	Humid	-0.555	0.62	68
82	DE-Wet	50.45	11.46	ENF	0.69	Humid	0.192	0.757	69
83	FI_Kaa	69.14	27.30	Wetland	0.75	Humid	-0.331	0.52	70
84	FI_Sod	67.36	26.64	ENF	0.70	Humid	-0.31	0.55	71
85	FR_Lq1	45.64	2.74	Grass	1.11	Humid	-0.175	0.888	22
86	FR_Lq2	45.64	2.74	Grass	1.09	Humid	-0.008	0.995	22
87	IE_Ca1	52.86	-6.92	Crop	0.73	Humid	0.036	0.997	72
88	IE_Dri	51.99	-8.75	Grass	0.88	Humid	0.965	0.168	73
89	IT_Non	44.69	11.09	DBF	0.72	Humid	-0.488	0.512	74
90	NL-Loo	52.17	5.74	ENF	0.68	Humid	0.108	0.752	75
91	SE-Deg	64.18	19.56	Grass	0.78	Humid	0.671	0.251	76
92	US_SP2	29.76	-82.24	Savanna	0.65	Humid	-0.252	0.63	62
93	US-Goo	34.25	-89.87	Grass	0.89	Humid	0.405	0.499	77
94	US-PFa	45.95	-90.27	MF	1.04	Humid	0.345	0.503	78
95	VU-Coc	-15.44	167.19	EBF	0.73	Humid	0.505	0.495	79

Section 3 Relative sensitivity of GPP and ET to drought

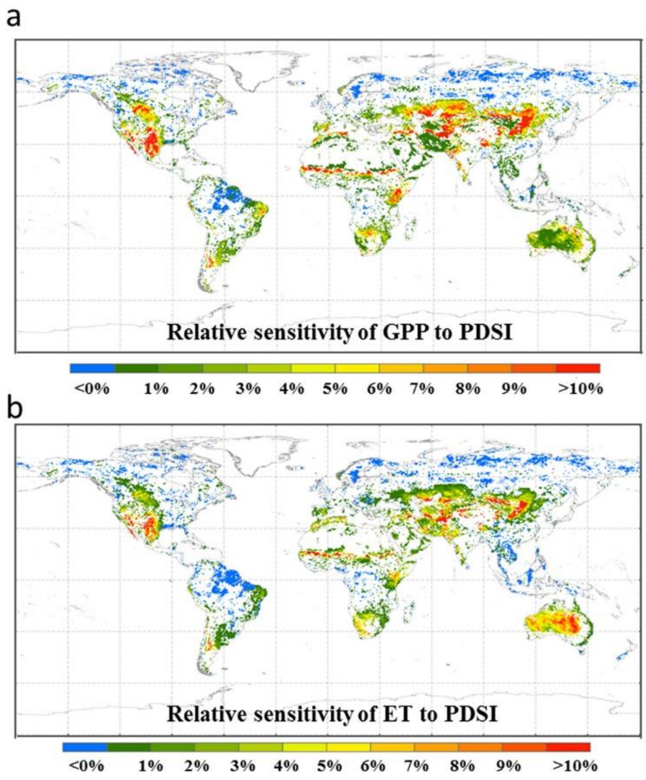

Supplementary Figure S6 Spatial distribution of relative sensitivity of annual (a) GPP and (b) ET to changes in PDSI based on detrended MTE data. Results show that the higher GPP sensitivity is generally located in global semi-arid and sub-humid regions (i.e., the Great Plain of North America, Northern Mexico, western part to the Pampas Steppe, south-eastern part to the Amazon Basin, mid-latitude of Euro-Asia, and the areas surrounding the Congo Basin) and higher ET sensitivity in global arid regions (i.e., the mid- and eastern Asia, south-eastern corner of Africa and majority of central- and western Australia). Our results also show a negative relationship between GPP (or ET) and PDSI in global energy-limited environments (i.e., tropical rainforests and boreal ecosystems). Maps were drawn using ArcMap 10.2.

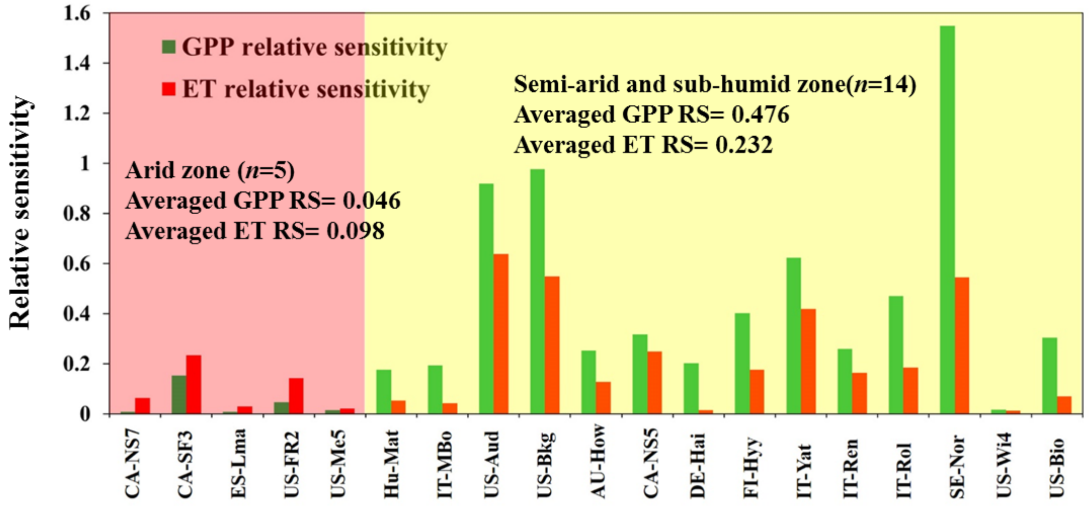

Supplementary Figure S7 Relative sensitivities of GPP and ET to changes in wetness index at 19 flux sites (site detail is provided in Table S1), at which the WUE-WI relationship are significant (*p*<0.1). Results show that the five arid sites have a higher ET sensitivity and the 14 sites located in semi-arid/sub-humid regions exhibit a higher GPP sensitivity. The background colours indicate different climate zones (red: arid zone; yellow: semi-arid/sub-humid zone). Image was drawn using Excel 2013.

Section 4. Memory effect of previous-year drought on ecosystem WUE

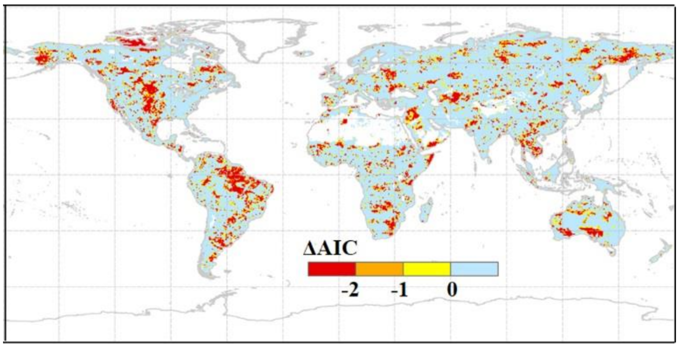

Supplementary Figure S8 Spatial distribution of the difference between the Akaike Information Criterion value (ΔAIC) of the WUE model using the two-year PDSI and that using the one-year PDSI. The new model (with two-year PDSI) is considered as an improvement over the old one (with one-year PDSI) if the AIC value reduced by more than 2.0 (i.e., ΔAIC< -2). Map was drawn using ArcMap 10.2.

References in the Supplementary Information

1. Feng, S. & Fu, Q. Expansion of global drylands under a warming climate. *Atmos. Chem. Phys*., **13**, 10081-10094 (2013).

2. Veenendaal, E. et al. Seasonal variation in energy fluxes and carbondioxide exchange for a broad-leaved semi-arid savanna (mopane woodland) in southern Africa. *Glob. Change Biol.* **10**, 318-328 (2004).

3. Goulden, M. et al. Nocturnal cold air drainage and pooling in a tropical forest. *J. Geophys. Res-Atmos.* **111**, D08S04 (2006).

4. Lafleur, P. et al. Interannual variability in the peatland-atmosphere carbon dioxide exchange at an ombrotrophic bog. *Glob. Biogeochem. Cy.* **17**, 1036 (2003).

5. Amiro, B. et al. Direct carbon emissions from Canadian forest fires, 1959-1999, Canadian Journal of Forest Research-Revue Canadienne De Recherche Foretiere **31**, 512-525 (2001).

6. Andreu, A. et al. Modelling surface energy fluxes over a Dehesa ecosystem using a two-source energy balance model. EGU General Assembly, Vienna, Austria (2013).

7. Heinsch, F. et al. Carbon dioxide exchange in a high marsh on the texas gulf coast: Effects of freshwater availability. *Agr Forest Meteorol.* **125**, 159-172 (2004).

8. Scott, R. et al. Effects of seasonal drought on net carbon dioxide exchange from a woody-plant-encroached semiarid grassland. *J. Geophys. Res-Biogeosci.* **114** (2009).

9. Scott, R. et al. Carbon dioxide exchange in a semidesert grassland through drought-induced vegetation change. *J. Geophys. Res-Biogeosci.* **115** (2010).

10. Law, B. et al. 2001. Carbon storage and fluxes in ponderosa pine forests at different developmental stages. *Glob. Change Biol.* **7**, 755-777 (2001).

11. Finnigan, J. & Leuning, R. Long term flux measurements-coordinate systems and averaging. In: Proceeding of the international workshop for advanced flux network and flux evaluation, center for global environmental research, national institute for environmental studies, Japan, Hokkaido, Japan, pp. 51-56. (2000).

12. Huete, L. et al. Evapotranspiration from Eucalypt open-forest savanna of Northern Australia. *Funct. Ecol.* **14**, 183-194 (2000).

13. Aubinet M, Chermanne B, Vandenhaute M, *et al.* (2001) Long term carbon dioxide exchange above a mixed forest in the Belgian Ardennes. *Agr. Forest Meteorol.* **108**, 293-315 (2000).

14. Dunn, A. et al. A long-term record of carbon exchange in a boreal black spruce forest: Means, responses to interannual variability, and decadal trends. *Glob Change Biol* **13**, 577-590 (2007).

15. Lafleur, P. et al. Interannual variability in the peatland-atmosphere carbon dioxide exchange at an ombrotrophic bog. *Glob Biogeochem Cy* **17**, Doi 10.1029/2002gb001983 (2003).

16. Giasson, M. et al. Ecosystem-level CO_2 fluxes from a boreal cutover in eastern Canada before and after scarification. *Agr Forest Meteorol* **140**, 23-40 (2006).

17. Anthoni, P. et al. Forest and agricultural land-use-dependent CO_2 exchange in Thuringia, Germany. *Glob Change Biol* **10**, 2005-2019 (2004).

18. Knohl, A. et al. Large carbon uptake by an unmanaged 250-year-old deciduous forest in Central Germany. *Agr. Forest Meteorol.* **118**, 151-167 (2003).

19. Owen, K. et al. Linking flux network measurements to continental scale simulations: ecosystem carbon dioxide exchange capacity under non-water-stressed conditions. *Global Change Biol.* **13**, 734-760 (2007).

20. Bernhofer, C. et al. The HartX-synthesis: an experimental approach to water and carbon exchange of a Scots pine plantation. *Theor. Appl. Climatol*. **53**, 173-183 (1996).

21. Sanz, M. et al. Effects of a dry and warm summer conditions on CO_2 and energy fluxes from three mediteranean ecosystems. *Geophys. Res. Abstr*. **6**, 3239 (2004).

22. Gilmanov, T. et al. Partitioning european grassland net ecosystem CO_2 exchange into gross primary productivity and ecosystem respiration using light response function analysis. *Agr Ecosyst Environ* **121**, 93-120 (2007).

23. Suni, T. et al. Long-term measurements of surface fluxes above a scots pine forest in Hyytiala, southern Finland, 1996-2001. *Boreal Environ Res* **8**, 287-301 (2003).

24. Berbigier, P. et al. CO_2 and water vapour fluxes for 2 years above Euroflux forest site. *Agr Forest Meteorol* **108**, 183-197 (2001).

25. Rambal, S. et al. The growth respiration component in eddy CO_2 flux from a Quercus Ilex Mediterranean forest. *Glob Change Biol* **10**, 1460-1469 (2004).

26. Granier, A. et al. The carbon balance of a young beech forest. *Funct. Ecol.* **14**, 312-325, (2000).

27. Nagy, Z. et al. Some preliminary results of the Hungarian grassland ecological research: carbon cycling and greenhourse gas balances under changing. *Cereal Res. Commun*. **33**, 279-281 (2005).

28. Grunzweig, J. et al. Carbon sequestration in arid-land forest. *Global Change Biol.* **9**, 791-799 (2003).

29. Maselli, F. et al. Use of remotely sensed and ancullary data for estimating forest gross primary productivity in Italy. *Remote Sens. Environ.* **100**, 563-575 (2006).

30. Migliavacca, M. et al. Seasonal and interannual patterns of carbon and water fluxes of a poplar plantation under peculiar eco-climatic conditions. *Agric. Forest Meteorol.* **149**, 1460-1476 (2009).

31. Montagnani, L.et al. A new mass conservation approach to the study of CO_2 advection in an alpine forest. *J Geophys Res-Atmos* **114**, doi 10.1029/2008jd010650 (2009).

32. Chiesi, M. et al. Modelling carbon budget of mediterranean forests using ground and remote sensing measurements. *Agr Forest Meteorol* **135**, 22-34, (2005).

33. Garbulsky, M. et al. Remote estimation of carbon dioxide uptake by a Mediterranean forest. *Glob Change Biol* **14**, 2860-2867 (2008).

34. Migliavacca, M. et al. Modeling gross primary production of agro-forestry ecosystems by assimilation of satellite-derived information in a process-based model. *Sensors-Basel* **9**, 922-942 (2009).

35. Rey, A. et al. Annual variation in soil respiration and its components in a coppice oak forest in central Italy. *Glob Change Biol* **8**, 851-866 (2009).

36. Tedeschi, V. et al. Soil respiration in a mediterranean oak forest at different developmental stages after coppicing. *Glob Change Biol* **12**, 110-121 (2006).

37. Jacobs, C. et al. Varibility of annual CO_2 exchange from Ducth grasslands. *Biogeosciences* **4**, 803-816 (2007).

38. Gӧckede, M. et al. Quality control of CarboEurope flux data – Part 1: Coupling footprint analyses with flux data quality assessment to evaluate sites in forest ecosystems. *Biogeosciences* **5**, 433-450 (2008).

39. Kurbatova, J. et al. Modeling carbon dynamics in two adjacent spruce forests with different soil conditions in Russia. *Biogeosciences* **5**, 969-980 (2008).

40. Lindroth, A. et al. Measurement of net ecosystem exchange, productivity and respiration in three spruce forests in sweden shows unexpectedly large soil carbon losses. *Biogeochemistry* **89**, 43-60 (2008).

41. Lagergren, F. et al. Biophysical controls on CO_2 fluxes of three Northern forests based on long-term eddy covariance data. *Tellus B* **60**, 143-152 (2008).

42. Groenendijk, M. et al. Assessing parameter variability in a photosynthesis model within and between plant functional types using global fluxnet eddy covariacne data. *Agr Forest Meteorol* **151**, 22-38 (2011).

43. Medlyn, B. et al. On the validation of models of forest CO_2 exchange using eddy covariance data: some perils and pitfalls. *Tree Physiol.* **25**, 839-857 (2005).

44. Noormets, A. et al. Age-dependent changes in ecosystem carbon fluxes in managed forests in northern Wisconsin, USA. *Ecosystems* **10**, 187-203 (2007).

45. Fischer, M. et al. Carbon, Water and Heat Flux Responses to Experimental Burning and Drought in a Tallgrass Prairie. *Agric. Forest Meteorol.* **166-167,** 169-174 (2012).

46. Yi, C. et al. Climate control of terrestrial carbon exchange across biomes and continents. *Environ Res Lett* **5**, 1-10 (2010).

47. Gilmanov, T. et al. Integration of CO_2 flux and remotely-sensed data for primary production and ecosystem respiration analyses in the northern Great Plains: Potential for quantitative spatial extrapolation. *Glob Ecol and Biogeography* **14**, 271-292 (2005).

48. Goldstein, A. et al. Effects of climate variability on the carbon dioxide, water and sensible heat fluxes above a ponderosa pine plantation in the Sierra Nevada (CA). *Agric. Forest Meteorol.* **101**, 113-129 (2000).

49. Meyers, T. & Hollinger, S. An assessment of storage terms in the surface energy balance of maize and soybean. *Agric. Forest Meteorol.* **125**, 105-115 (2004).

50. Urbanski, S.et al. Factors controlling CO_2 exchange on timescales from hourly to decadal at Harvard forest. *J Geophys Res-Biogeo* **112**, G02020 (2007).

51. Hollinger, D. et al. Spatial and temporal variability in forest-atmosphere CO_2 exchange. *Glob Change Biol* **10**, 1689-1706 (2004).

52. Sun, O. et al. Dynamics of carbon stocks in soils and detritus across chronosequences of different forest types in the pacific northwest, USA. *Glob Change Biol* **10**, 1470-1481 (2004).

53. Schmid, H. et al. Measurements of CO_2 and energy fluxes over a mixed hardwood forest in the mid-western united states. *Agr Forest Meteorol* **103**, 357-374 (2000).

54. Verma, S. et al. Annual carbon dioxide exchange in irrigated and rainfed maize-based agroecosysems. *Agric. Forest Meteorol.* **131**, 77-96 (2005).

55. Ma, S. et al. Inter-annual variability in carbon dioxide exchange of an oak/grass savanna and open grassland in California. *Agric. Forest Meteorol.* **147**, 157-171 (2007).

56. Gough, C. et al. Multi-year convergence of biometric and meteorological estimates of forest carbon storage. *Agr Forest Meteorol* 148, 158-170 (2008).

57. Cook, B. et al. Carbon exchange and venting anomalies in an upland deciduous forest in Northern Wisconsin, USA. *Agr Forest Meteorol* **126**, 271-295 (2004).

58. Jenkins, J. et al. Refining light-use efficiency calculations for a deciduous forest canopy using simultaneous tower-based carbon flux and radiometric measurements. *Agr Forest Meteorol* **143**, 64-79 (2007).

59. Allison, V. et al. Changes in soil microbial community structure in a tallgrass prairie chronosequence. *Soil Sci. Soc. Am. J.* **69**, 1412-1421 (2005).

60. Monson, R. et al. Carbon sequestration in a high-elevation, subalpine forest. *Glob Change Biol* **8**, 459-478 (2002).

61. Clark, K. et al. Environmental controls over net exchanges of carbon dioxide from contrasting florida ecosystems. *Ecol. Appl*. **9**, 936-948 (1999).

62. Griffis, T. et al. Direct measurement of biosphere-atmosphere isotopic CO_2 exchange using the eddy covariance technique. *J. Geophys. Res.* **113**(D8) (2008).

63. Lipson, D. et al. Effects of elevated atmospheric CO*2* on soil microbial biomass, activity, and diversity in a chaparral ecosystem. *Appl. Environ. Microbiol.* **71**, 8573-8580 (2005)

64. Desai, A. et al. Comparing net ecosystem exchange of carbon dioxide between an old-growth and mature forest in the upper Midwest, USA. *Agric. Forest. Meteorol.* **128**, 33-55 (2005).

65. Wohlfahrt, G. et al. Seasonal and inter-annual variability of the net ecosystem CO_2 exchange of a temperate mountain grassland: Effects of weather and management. *J Geophys Res-Atmos* **113**, doi:10.1029/2007/JD009286 (2008).

66. Carrara, A. et al. Seasonal changes in photosynthesis, respiration and NEE of a mixed temperate forest. *Agr Forest Meteorol* **126**, 15-31 (2004).

67. Ammann, C. et al. The carbon budget of newly established temperate grassland depends on management intensity. *Agr Ecosyst Environ* **121**, 5-20 (2007).

68. Don, A. et al. Impact of afforestation-associated management changes on the carbon balance of grassland. *Glob Change Biol* **15**, 1990-2002 (2009).

69. Rebmann C. et al. Treatment and assessment of the CO_2-exchange at a complex forest site in Thuringia, Germany. *Agr Forest Meteorol* **150**, 684-691 (2010).

70. Aurela, M. et al. Annual CO_2 balance of a subarctic fen in northern Europe: importance of the wintertime efflux. *J. Geophys. Res.* **107**, 4607 (2002).

71. Thum, T. Parameterization of two photosynthesis models at the canopy scale in a northern boreal Scots pine forest. *Tellus B* **59**, 874-890 (2007).

72. Black, T. et al. Long-term trend in solar irriadiance in Ireland and their potential effects on gross primary productivity. *Agric. Forest Meteorol.* **141**, 118-132 (2006).

73. Peichl, M. et al. Six-year stable annual uptake of carbon dioxide in intensively managed humid temperate grassland. *Ecosystems* **14**, 112-126 (2011).

74. Nordino, M. et al. Primary productivity and evapotranspiration of a mixed forest. In: Congress CNR-I.S.A. Fo, Istituto per I Sistemi Agricoli e Forestali del Mediteeranco, Portici, Italy (2002).

75. Dolman, A. et al. The carbon uptake of a mid latitude pine forest growing on sandy soil. *Agr Forest Meteorol* **111**,157-170 (2002).

76. Sagerfors, J. et al. Annual CO_2 exchange between a nutrient-poor, minerotrophic, boreal mire and the atmosphere. *J Geophys Res-Biogeo* **113**, DOI: 10.1029/2006JG000306 (2008).

77. Wilson, T., & Meyers, T. Determining vegetation indices from solar and photosynthetically active radiation fluxes. *Agric. Forest Meteorol.* **144**, 160-179 (2007).

78. Davis, K. et al. The annual cycles of CO_2 and H_2O exchange over a northern mixed forest as observed from a very tall tower. *Glob Change Biol* **9**, 1278-1293 (2003).

79. Fisher, J. et al. The land-atmosphere water flux in the tropics. *Global Change Biol.* 15, 2694-2714 (2009).
